# An inducible human LDLR transgenic mouse model for enhanced lentiviral gene delivery to murine immune cells

**DOI:** 10.3389/fimmu.2026.1941021

**Published:** 2026-10-07

**Authors:** Jian Li, Beatriz Goncalves, Gabrielle Melamed, Dillon O’Neill, Ryan Cozzone, Sara Iuliucci, Austin Cozzone, Mohamad-Gabriel Alameh, Leyuan Ma

**Affiliations:** 1 The Raymond G. Perelman Center for Cellular and Molecular Therapeutics, Children’s Hospital of Philadelphia, Philadelphia, PA, United States; 2 Department of Cancer Biology, Perelman School of Medicine, University of Pennsylvania, Philadelphia, PA, United States; 3 Department of Experimental Pathology, Children’s Hospital of Philadelphia, Philadelphia, PA, United States; 4 Department of Pathology and Laboratory Medicine, Perelman School of Medicine, University of Pennsylvania, Philadelphia, PA, United States; 5 Institute of RNA Innovation, University of Pennsylvania, Philadelphia, PA, United States; 6 Center for Cellular Immunotherapies, University of Pennsylvania, Philadelphia, PA, United States; 7 Abramson Cancer Center, University of Pennsylvania, Philadelphia, PA, United States

**Keywords:** Cre recombinase, hLDLR, lentivirus, mRNA-LNP, transgenic mouse, VSV-G

## Abstract

Lentivirus (LV)-based manipulation of immune cells is a commonly used approach in cell therapies. However, the most frequently used Vesicular Stomatitis Virus Glycoprotein (VSV-G) pseudotyping approach is inefficient to support LV gene delivery of mouse immune cells due to the lack of an optimal entry receptor. Here, we present a transgenic mouse model with Cre recombinase-inducible expression of the human Low Density Lipoprotein Receptor (hLDLR), the natural entry receptor of VSV-G LV. Surprisingly, we found that Cre induces robust hLDLR expression across multiple immune cells only under activation conditions. Induction of hLDLR expression by genetic crossing with Cre-transgenic mice or exposure to Cre mRNA-lipid nanoparticles (LNPs) potently sensitized murine immune cells, especially T cells, to VSV-G LV-mediated gene delivery. This novel transgenic mouse model serves as a valuable tool for investigating immune cell function and engineered cell therapies for cancer in syngeneic mouse models.

## Introduction

Efficient genetic manipulation of mouse immune cells, including T-cells, is critical to accurately investigate new immunotherapies as well as to interrogate the mechanism of existing therapies in immunocompetent mouse models (1, 2). A number of methods have been used for genetic engineering of mouse immune cells, especially T cells, such as viral vectors (3), electroporation (4), and lipid nanoparticles (5). Among these methods, lentivirus (LV) transduction has been the most widely used method, given its broad availability and genomic integration for a stable phenotype (6). Vesicular Stomatitis Virus Glycoprotein (VSV-G) pseudotyped lentiviruses are the most frequently and widely used LVs in the research setting (7). However, VSV-G LV has an inherently low efficiency of transducing mouse immune cells, hindering immunological studies in the syngeneic mouse models (8, 9). For example, previous studies have reported low levels of transgene expression following lentiviral transduction of primary mouse T cells in the absence of transduction enhancement reagents (10).

Low-density lipoprotein receptor (LDLR) was found to be the predominant entry receptor for VSV-G LVs (11). Binding of VSV-G to the LDLR and the LDLR family of receptors allows the lentivirus to attach to the cell and triggers entry into the target cell via clathrin-dependent receptor-mediated endocytosis (12). In addition to facilitating LV attachment to the host cells, VSV-G also functions as a fusogen, mediating fusion of the viral and cellular membranes and viral genome delivery into the cytosol. Despite the homology and functional conservation between the mouse and human LDLR and their expression on T cells (11, 13), the observation that VSV-G LV exhibited a lower capability in transducing murine T cells suggested a more efficient role of human LDLR (hLDLR) in engaging VSV-G and promoting VSV-G LV gene delivery.

Here, we generated a hLDLR transgenic mouse model by inserting a Cre-inducible hLDLR expression cassette into the Rosa26 locus. By crossing the Rosa26-hLDLR mice with a Cre recombinase-carrying mice or transient Cre recombinase delivery using mRNA-LNPs, we observed high-magnitude and robust hLDLR induction on various immune cells following *ex vivo* stimulation. As expected, the expression of hLDLR on mouse T-cells increased virus-cell interactions as well as VSV-G LV gene delivery, with a strong correlation between hLDLR expression and transgene expression.

## Results

### Ectopic hLDLR expression is sufficient to promote VSV-G LV gene delivery to murine T cells

It was suspected that mechanisms such as poor virus binding, impaired reverse transcription and nuclear import of the viral pre-integration complex could contribute to poor LV gene delivery into murine T cells (9, 14). We hypothesized that ectopic hLDLR expression in murine T cells could improve VSV-G LV binding and gene delivery to murine T cells (**Figure 1A**). Given that murine T cells are highly susceptible to retrovirus, we first transduced hLDLR into activated mouse T cells using retrovirus, followed 24 hours later with a second round of gene delivery using VSV-G LV with green fluorescent protein (GFP) as a reporter. Next, we used flow cytometry to sort the untransduced and LDLR^high^ T cell population and compared the efficiency of VSV-G LV gene delivery (**Figure 1B**). Notably, VSV-G LV efficiently delivered the reporter gene GFP into hLDLR^high^ mouse T cells (**Figure 1B**). These results suggest that ectopic hLDLR expression in murine T cells is sufficient to promote VSV-G LV gene delivery.

**Figure 1:**
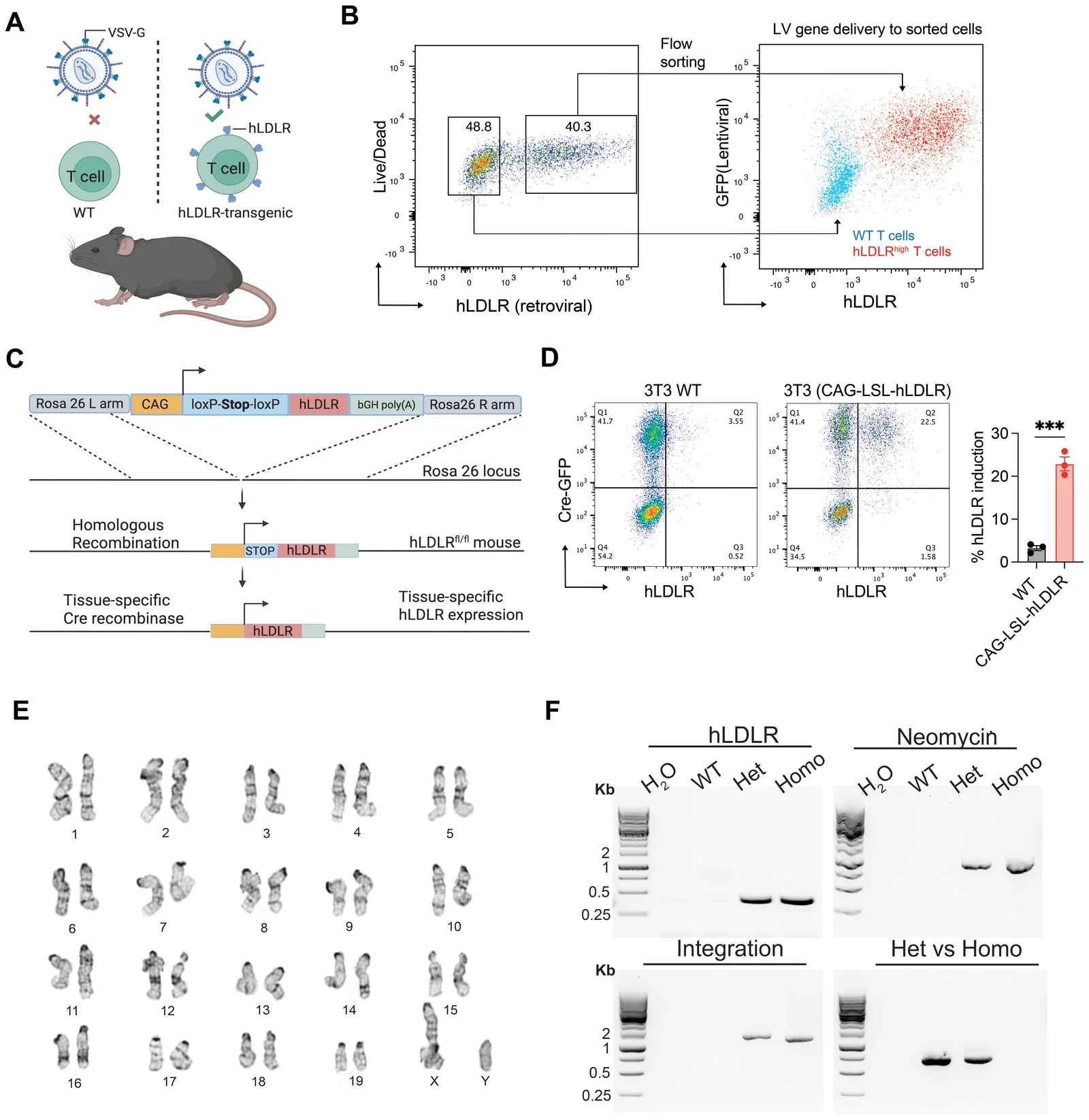
Establishment of the hLDLR-transgenic mouse. **(A)** Schematics showing hLDLR-transgene as the VSV-G LV entry receptor on murine T cells. Schematics were generated using BioRender. **(B)** Ectopic expression of hLDLR using retrovirus in murine T cells promotes VSV-G LV gene delivery. GFP is a reporter gene in the retroviral vector. LDLR^null^ (WT) and LDLR^high^ populations were flow sorted and then received LV (GFP) gene delivery. **(C)** Design of the Rosa26 locus targeting CAG-LSL-hLDLR targeting vector and induction of hLDLR in a tissue-specific manner by crossing with mice carrying tissue-specific Cre recombinase. **(D)** *In vitro* validation of the targeting vectors and Cre-mediated hLDLR induction in NIH 3T3 cells. The targeting vector was linearized and then transiently transfected into 3T3 cells followed by neomycin selection. Fully selected cells were then transduced with a retroviral vector carrying a Cre-GFP fusion protein. Cells were monitored for GFP and hLDLR expression using flow cytometry 48 hours later. **(E)** Karyotyping of the validated 1C3 ES clone. **(F)** Genotyping of fully established hLDLR-transgenic mice using PCRs. PCR primers were designed for major components of the targeting vector including, hLDLR, neomycin, as well as for testing integration into the Rosa26 locus and for distinguishing homozygous and heterozygous mice. See **Supplementary Figure 1** for details. Error bars represent the standard error, ***p<0.001, by Student’s t-test.

### The generation of a hLDLR -transgenic mouse line

To create a sustainable solution to enable robust and efficient VSV-LV gene delivery into murine T cells and potentially other refractory murine cell types, we sought to develop a transgenic mouse line permitting ectopic hLDLR expression in desired cell types. To this end, the hLDLR gene was cloned into a ROSA26-allele targeting vector for stable integration into the ROSA26 genomic locus, a safe harbor that is commonly used for robust transgene expression (15) (**Figure 1C**). To ensure high-level transgene expression, a strong synthetic promoter, cytomegalovirus (CMV) enhancer/chicken β-actin chimeric (CAG) promoter (16), was inserted upstream of the hLDLR open reading frame (ORF) (**Figure 1C**). A LoxP-STOP-LoxP cassette (LSL) was placed between the CAG promoter and the hLDLR ORF, permitting Cre-induced excision and restoration of hLDLR expression (**Figure 1C**). A PGK-neomycin cassette was included within the LoxP-STOP-LoxP cassette to facilitate the positive selection of cell clones that had successful recombination. To characterize the targeting vector *in vitro*, we transfected NIH 3T3 cells with the linearized Rosa26-CAG-LSL-hLDLR-targeting vector, followed by stable selection with neomycin. Then we transduced the selected population with a retrovirus carrying Cre-GFP. Expression of Cre led to successful hLDLR induction on the surface of 3T3 cells (**Figure 1D**).

The Rosa26-hLDLR targeting vector was then transfected into C57BL/6N JM8A3 ES cells (17) and selected as previously described (18). After screening 18 clones, we found that ES clone 1C3 expanded well and possessed successful integration of the whole targeting CAG-LSL-hLDLR cassette. Cytogenetic analysis confirmed a normal karyotype (**Figure 1E**). ES cells from clone 1C3 were microinjected into blastocysts from C57BL/6J mice. Pups were genotyped for the presence of the CAG-LSL-hLDLR cassette (**Figure 1F**; **Supplementary Figure S1**) and then backcrossed over 10 generations to C57BL/6J mice. Both the hLDLR homozygous strain (hLDLR^fl/fl^) and heterozygous (hLDLR^fl/-^) strain are fertile.

### Cre-inducible hLDLR expression in immune cells after activation

To characterize Cre inducible hLDLR expression, we bred the hLDLR^fl/fl^ homozygous strain with CMV-Cre^+/+^ homozygous strain to generate F1 hybrid LDLR^fl/-^;Cre^+/-^ mice (**Figure 2A**). Immunophenotyping showed normal immune cell development in the hLDLR^fl/fl^ homozygous and F1 hybrid mice (**Figure 2B**). Interestingly, hLDLR^fl/fl^ homozygous mice and the F1 hybrid LDLR^fl/-^;Cre^+/-^ mice exhibited mildly but significantly increased T cells and NK cells (**Figure 2B**).

**Figure 2:**
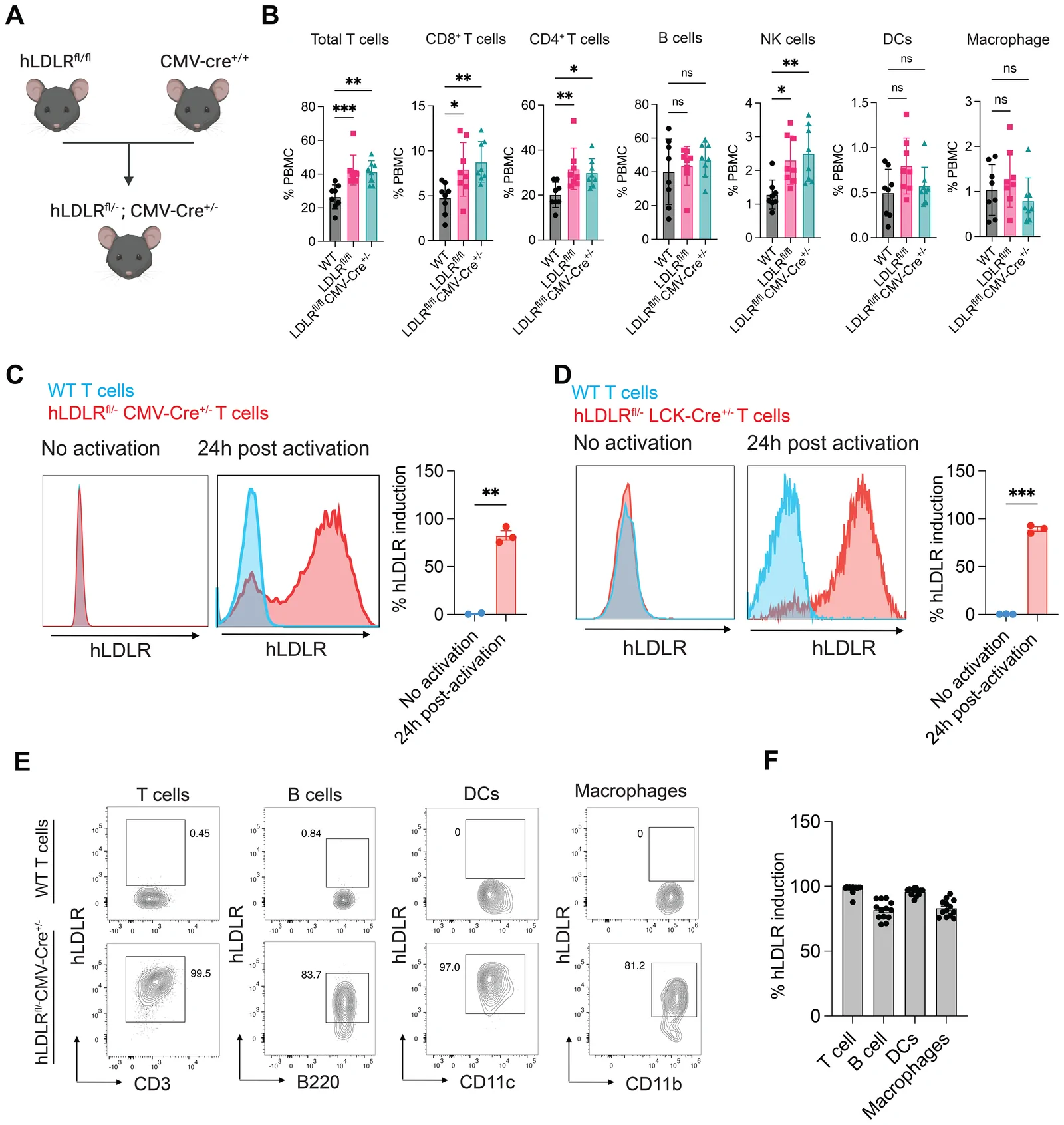
Characterization of Cre-mediated hLDLR induction in transgenic mice. **(A)** Schematics showing the crossing of hLDLR^fl/fl^ mice with Cre-carrying mice. We used CMV-Cre^+/+^ female in this case to ensure all F1 hybrids harbor both the hLDLR and Cre alleles. Schematics were generated using BioRender. **(B)** Characterization of the proportion of the major immune cell populations in the peripheral blood of WT, hLDLR^fl/fl^ and hLDLR^fl/-^; CMV-Cre^+/-^ mice. Each dot represents an individual mouse. **(C)** Monitoring hLDLR induction in murine T cells from hLDLR^fl/-^; CMV-Cre^+/-^ mice before and after 24 hours of activation. **(D)** Monitoring hLDLR induction in murine T cells from hLDLR^fl/-^; LCK-Cre^+/-^ mice before and after 24 hours of activation. **(E)** hLDLR induction in representative peripheral blood immune cells in WT and hLDLR^fl/-^; CMV-Cre^+/-^ murine T cells after 24 hours of activation. **(E)** Summary of hLDLR induction in activated peripheral blood immune cells from WT and hLDLR^fl/-^; CMV-Cre^+/-^ mice. Each dot represents an individual mouse. Error bars represent the standard error. *p<0.05, **p<0.01, ***p<0.001, ns, non-significant by one-way ANOVA for B, and by Student’s t-test for **(C, D)**.

Surprisingly, there is minimal hLDLR expression on naïve T cells (**Figure 2C**, left panel). To rule out the possibility of poor Cre expression in murine T cells due to CMV promoter silencing (15), we bred the hLDLR homozygous strain with LCK-Cre transgenic mice. We also observed the lack of LDLR expression in naïve T cells (**Figure 2D**, left panel). To test if the activation status of T cells affects LDLR induction, we stimulated naïve T cells with αCD3/CD28 antibody-coated plate for 24 hours and observed marked induction of hLDLR expression in both LDLR^fl/-^;CMV-Cre^+/-^ and LDLR^fl/-^;LCK-Cre^+/-^ T cells (**Figures 2C, D**, right panels).

Likewise, after activation of whole peripheral blood mononuclear cells (PBMCs), hLDLR expression level was markedly induced in multiple immune cell populations, including T cells, B cells, DCs, and macrophages (**Figure 2E**), with > 90% induction in DCs and T cells and >80% induction in B cells and macrophages (**Figure 2F**). Unexpectedly, hLDLR induction in T cells seems to be influenced by the activation time. The peak expression was achieved 24–72 hours post-activation, followed by reduction of hLDLR expression after 72 hours of activation (**Supplementary Figure S2**), which likely associates with dampened T cell activation, decreased metabolism and overall biosynthesis (19, 20).

### hLDLR induction improved VSV-G LV gene delivery to murine immune cells

Next, we sought to determine if the transgenic hLDLR expression in murine immune cells could improve VSV-G LV binding (**Figure 3A**). Focusing on T cells, we observed robust VSV-G LV binding to activated LDLR^fl/-^;CMV-Cre^+/-^ murine T cells exhibiting hLDLR expression but not WT T cells (**Figure 3A**). We next tested if the transgenic hLDLR expression could facilitate VSV-G LV gene delivery. Splenocytes from F1 hybrid LDLR^fl/-^;CMV-Cre^+/-^ or control WT mice were pre-stimulated overnight and then received VSV-G LV gene delivery with a blue fluorescent protein (BFP) as a reporter. As expected, only the splenocytes from F1 hybrid mice had successful reporter gene expression (**Figures 3B, C**).

**Figure 3:**
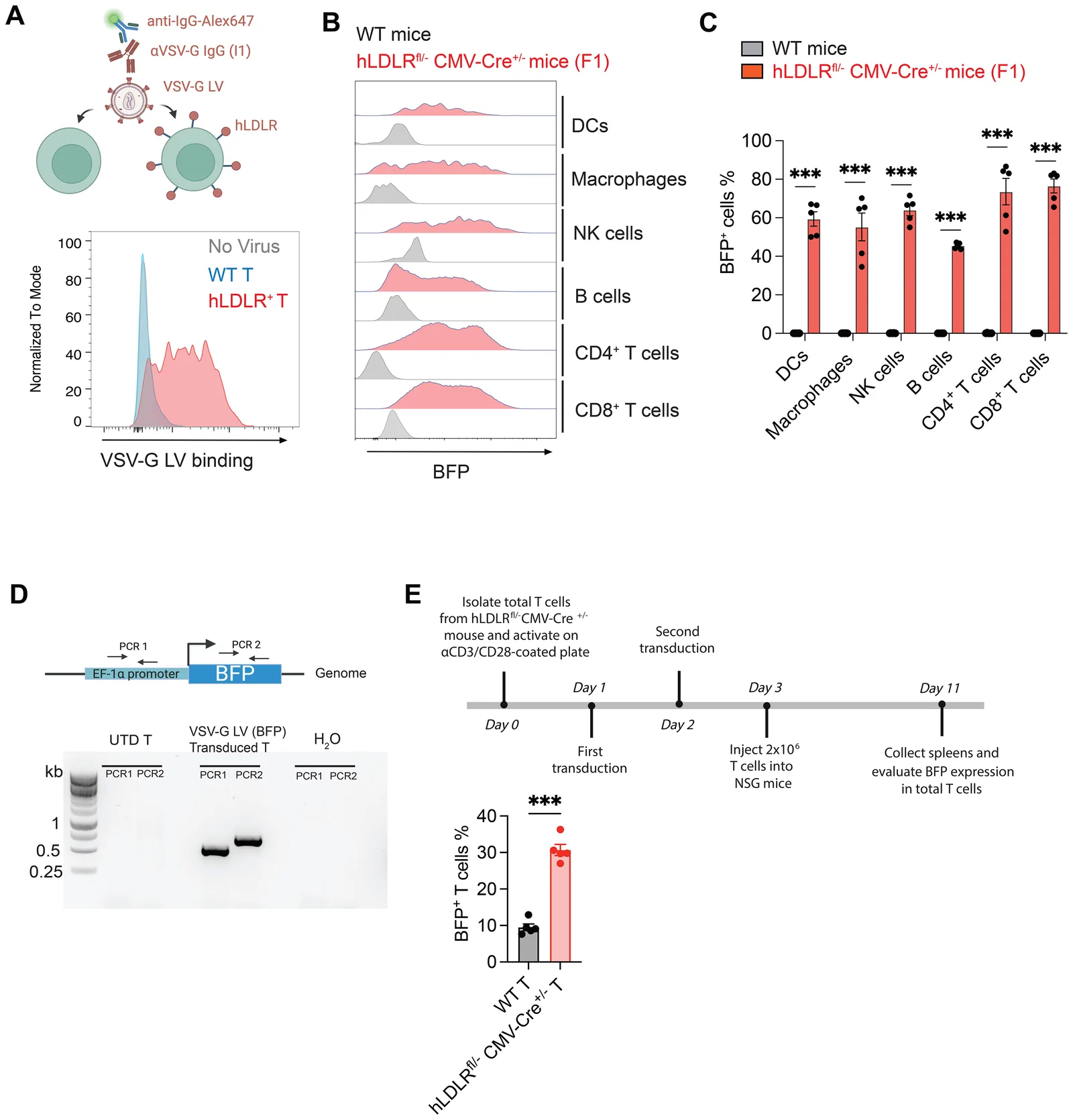
hLDLR induction facilitates VSV-G LV binding and gene delivery to murine immune cells. **(A)** Flow cytometry analysis of VSV-G LV binding to murine T cells with or without hLDLR induction. An anti-VSV-G antibody clone I1 was used for detecting VSV-G LVs. Schematics were generated using BioRender. **(B)** Representative histogram showing VSV-G LV gene delivery to WT or hLDLR^fl/-^; CMV-Cre^+/-^ murine immune cells post activation. BFP is used as the reporter gene to monitor gene delivery. **(C)** Summary of BFP expression in various splenic immune cells from hLDLR^fl/-^; CMV-Cre^+/-^ mice in **(B)**. **(D)** Characterization of the stable integration of VSV-G LV (BFP) into the murine T cell genome. PCR primers were designed to amplify both the EF1α promoter (PCR1) and BFP (PCR2) from purified genomic DNA from untransduced (UTD) or VSV-G LV-transduced hLDLR^fl/-^; CMV-Cre^+/-^ T cells. **(E)** Validation of transgene expression *in vivo*. Following the depicted timeline, activated WT or hLDLR^fl/-^; CMV-Cre^+/-^ T cells received two rounds of VSV-G LV (BFP) gene delivery and then were transferred into NSG mice on day 3. On day 11, spleens were collected and analyzed for BFP expression in T cells. Each dot represents an individual mouse (n=5). Error bars represent the standard error, ***p<0.01, by Student’s t-test for **(C, E)**.

We also confirmed stable integration of LV genome into murine T cells with clear PCR detection of the promoter EF-1α and reporter gene BFP in the extracted genomic DNA from LDLR^fl/-^;CMV-Cre^+/-^ T cells receiving VSV-G LV gene delivery (**Figure 3D**). To further test the stability of transgene expression *in vivo*, we transferred LDLR^fl/-^;CMV-Cre^+/-^ T cells 24 hours post gene delivery into immunodeficient NSG mice, to allow easy detection of adoptively transferred T cells while avoiding rejection due to the potential combined immunogenicity of hLDLR and Cre (21) in the immunocompetent mice (**Figure 3E**). Seven days post adoptive transfer, the reporter BFP expression could be detected in around 30% of LDLR^fl/-^;CMV-Cre^+/-^ T cells compared to ~10% in WT T cells (**Figure 3E**). Finally, we showed that VSV-G LV could deliver human IL2, a cytokine with well-established human and mouse cross-reactivity (22), into LDLR^fl/-^;CMV-Cre^+/-^ mouse T cells (**Supplementary Figure S3**), confirming the feasibility of this system for expressing a therapeutically relevant payload.

### Cre mRNA-LNP as an efficient alternative for inducing hLDLR expression to promote VSV-G LV gene delivery

Given the lengthy time required for mouse breeding and F1 hybrid production, we next tested if the Cre recombinase could be delivered to specific immune cells to induce hLDLR expression using mRNA-LNPs modified with an immune cell-specific targeting antibody (23–25) (**Figure 4A**). We previously found that αCD5-conjugated mRNA LNPs could efficiently deliver mRNA payload to T cells (26). Therefore, we focused on αCD5-conjugated mRNA LNPs to maximize the Cre mRNA delivery to T cells (**Supplementary Figures S4A, B**). Indeed, pulsing αCD5 Cre mRNA-LNP into bulk activated hLDLR^fl/fl^ splenocytes preferentially induced hLDLR expression in T cells in 24 hours (**Figures 4B, C**). After transient Cre mRNA-LNP treatment of CD45.2^+^ hLDLR^fl/fl^ T cells and hLDLR induction, we adoptively transferred ~2x10^6^ treated or control non-treated T cells into CD45.1^+^ recipient mice. A week later, flow cytometry analysis demonstrated comparable CD45.2^+^ T cell levels in both groups, suggesting minimal immunogenicity of hLDLR (**Supplementary Figure S5**).

**Figure 4:**
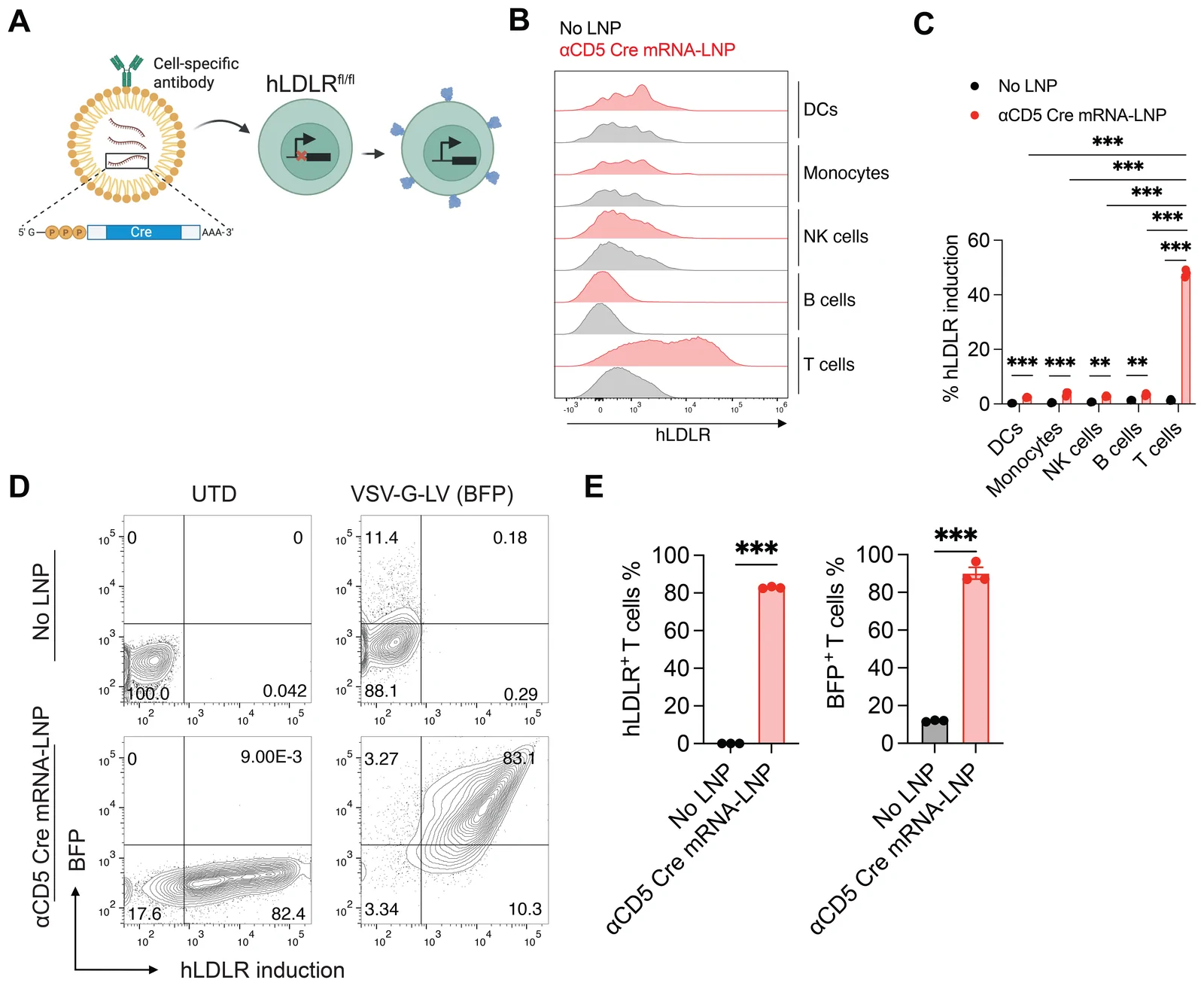
Targeted mRNA-LNP mediated Cre delivery as an efficient method for inducing hLDLR expression in murine T cells. **(A)** Schematics showing the design of targeted Cre mRNA-LNP for cell-type-specific induction of hLDLR. Schematics were generated using BioRender. **(B)** Characterization of CD5-targeted Cre mRNA-LNP for selective hLDLR induction in murine T cells *in vitro*. Murine splenocytes from hLDLR^fl/fl^ mouse were pulsed with CD5-targeted Cre mRNA-LNP followed by 24 hours of activation and then monitored for hLDLR induction using flow cytometry. **(C)** Summary of hLDLR induction in various splenic immune cells after treatment with CD5-targeted Cre mRNA-LNP in **(B)**. **(D)** Representative flow cytometry plots showing hLDLR induction and VSV-G LV (BFP) gene delivery into hLDLR^fl/fl^ T cells with or without prior Cre mRNA-LNP treatment on day 4, 48 hours after LV gene delivery following the same setup as depicted in **Figure 3E**. **(E)** Summary plots showing hLDLR induction and VSV-G LV (BFP) gene delivery into hLDLR^fl/fl^ T cells with or without Cre mRNA-LNP treatment in **(D)** Unless otherwise stated, all LNP treatments in this figure were performed using 1μg(mRNA-equivalent)/million cells. Each dot represents an individual treatment (n=3). Error bars represent the standard error, **p<0.01, ***p<0.001 by Student’s t-test for **(C, E)**.

Using purified hLDLR^fl/fl^ T cells, we further showed VSV-G LV gene delivery only occurred in T cells pre-treated with Cre-mRNA LNPs and the gene delivery efficiency strongly correlates with the hLDLR induction level in T cells (**Figure 4D**), achieving ~80% overall hLDLR induction and ~90% gene delivery efficiency (**Figure 4E**).

## Discussion

In this study, we developed and characterized an inducible hLDLR knock-in mouse line to overcome the challenge of inefficient VSV-G-pseudotyped LV gene delivery to murine immune cells. By targeting a Cre-inducible CAG-LSL-hLDLR cassette to the Rosa26 locus, we demonstrate that high-level hLDLR expression is both necessary and sufficient to bridge VSV-G–LV binding and robust gene delivery into mouse immune cells. We achieved robust induction of hLDLR on multiple immune cell subsets, including T cells, B cells, DCs, and macrophages, and converted otherwise refractory murine immune cells into suitable targets for the broadly used VSV-G LVs.

A notable feature of the Rosa26-CAG-LSL-hLDLR mouse line is the tight coupling of hLDLR expression to immune cell activation. Naïve T cells do not express a detectable level of hLDLR as monitored by flow cytometry, whereas a 24-hour T cell stimulation robustly induced a high-level hLDLR expression. Unexpectedly, following the peak induction at 24 hours of T cell activation, hLDLR expression decreased after 72 hours, correlating with dampening T cell activation. This activation-linked regulation has practical implications: it defines a temporal window of 24–72 hours during which ex vivo VSV-G LV gene delivery can be efficiently achieved. This finding is unexpected because the Rosa26 locus itself is broadly permissive and widely used as a “safe harbor” for ubiquitous transgene expression in mice (15), and the CAG synthetic promoter was known for its robustness and potency in driving high-level transgene expression across murine cells (16), including immune cells. In our system, the apparent activation-dependent hLDLR expression may reflect the combined effects of the Cre-inducible CAG-LSL-hLDLR cassette at the Rosa 26 locus and the intrinsic biology of T cell activation - namely, global upregulation of biosynthetic and trafficking programs. Future studies quantifying hLDLR transcription and Rosa 26 locus accessibility in naïve versus activated T cells or other immune cell types will help dissect the relative contributions of locus activity, promoter strength, and activation-induced changes in transgene processing.

While our *in vitro* and *in vivo* data demonstrated successful gene delivery by VSV-G LV to hLDLR^+^ murine T cells, we observed a decrease in the transgene-expressing cells after adoptive cell transfer *in vivo*. It is possible that murine T cells after gene delivery using non-optimized LV formulation were under cellular stress and susceptible to cell death or reduced fitness (27, 28). We also cannot rule out other factors that might contribute to LV gene delivery into murine T cells. It has been reported that persistent post-entry barriers, such as reverse transcription, nuclear import, integration site selection, or chromatin-level regulation of transgene expression (9, 14), likely remain operative and will require additional engineering to fully align murine T cells with their human counterparts, which is beyond the scope of this work.

Finally, we show that hLDLR induction can be achieved via either genetic crossing to a Cre recombinase-bearing strain or transient delivery of Cre recombinase using mRNA-LNPs. While the genetic approach offers stable, compartment-selective, and heritable control of hLDLR induction for long-term studies, the mRNA–LNP strategy provides rapid, strain-agnostic induction suitable for *ex vivo* manipulation. In particular, the conjugation of an antibody targeting a cell type-specific membrane protein, in this case CD5 for T cells, to mRNA-LNPs permits cell-type-specific targeting and hLDLR induction, with a potential for precision *in vivo* perturbations in the future (23–25). Together, these complementary modalities position the hLDLR transgenic mouse line as a versatile tool for VSV-G LV-mediated gene manipulation of murine immune cells in syngeneic tumor models.

### Limitations and future directions

Several limitations of this approach warrant consideration. First, the dependence of hLDLR induction on activation status creates a narrow temporal window for efficient gene delivery and may complicate *in vivo* applications where the kinetics and localization of T-cell activation are heterogeneous and difficult to control and would require in-depth characterization. Second, each Cre delivery strategy carries practical constraints. Genetic crossing to Cre driver lines is time-consuming, limits flexibility when multiple strains or disease models are required and may introduce confounding phenotypes from the Cre line itself. Conversely, the Cre mRNA-LNP approaches add formulation complexity and may exhibit variable transfection efficiency across immune subsets in an *in vivo* delivery setting. These factors should be taken into account when extrapolating this platform to different experimental settings or disease models. Third, the system is optimized for VSV-G pseudotyped LV and may not directly address barriers related to other viral vectors or envelope proteins. Future work integrating this entry-enhanced platform with self-inactivating LV designs, additional modulation of post-entry restrictions will be important to fully exploit the potential of this transgenic line for murine T-cell engineering and cancer immunotherapy research.

## Materials and methods

### Cell lines and cell culture

HEK293T cells were kindly provided by the research vector core at the Children’s Hospital of Philadelphia (CHOP) and maintained in high-glucose Dulbecco’s Modified Eagle’s Medium (DMEM) (VWR, cat #16777-200) with 10% fetal bovine serum (FBS) (Fischer Scientific, cat #MT35010CV) and 1% Penicillin-Streptomycin (ThermoFischer, cat #15140122). NIH 3T3 cells and Jurkat cells were obtained from ATCC and maintained in Roswell Park Memorial Institute (RPMI) 1640 medium (cat #SH30255FS) supplemented with 10% FBS and 1% Penicillin-Streptomycin.

### Rosa26-hLDLR chimera line generation

C57BL/6N JM8A3 ES cells (Pettitt, S.J., et al, Agouti C57BL/6N embryonic stem cells for mouse genetic resources. Nature Methods 6, 493-495, 2009) were electroporated with 35ug of linearized targeting vector, and cultured on irradiated MEF feeder cells according to the MMRRC Protocol for Culturing JM8 Clones. Targeted clones were selected using 300ug/mL Geneticin (Gibco) for 6 days and then isolated. Cytogenic analysis was performed by Cell Line Genetics (Madison, WI) and targeted clone 1C3 was found to have an apparently normal mouse male karyotype. Cells from clone 1C3 were microinjected into blastocysts from C57BL/6J mice (Jackson Laboratories, Bar Harbor, ME). Seventeen of the 35 pups were chimeric and 7 were male with at least 90% ES cell contribution to coat color. These were bred for germline transmission of the targeted allele. Mice were housed in fully AAALAC-accredited facilities and used in accordance with protocols approved by the IACUC at the Massachusetts Institute of Technology or the Children’s Hospital of Philadelphia (CHOP), respectively.

### Mouse strain breeding and maintenance

The NSG mice, CMV-Cre stain and LCK-Cre stain were purchased from the Jackson Laboratory. Two genetically modified mouse strains were maintained for this study: homozygous human low-density lipoprotein receptor-transgenic (hLDLR^fl/fl^) mice and LDLR^fl/-^ mice carrying the Cre transgene (LDLR^fl/-^; Cre^+/-^). Both strains were maintained on a C57BL/6 genetic background. Breeding colonies were established and maintained in-house at the CHOP animal facility. All animal procedures were conducted in accordance with institutional standard operating procedures and approved by the CHOP Institutional Animal Care and Use Committee (IACUC; Protocol No. 22-001447). Animal health and welfare were monitored in collaboration with the Department of Veterinary Resources (DVR).

The primary colony consisted of LDLR^fl/fl^ and LDLR^fl/-^ mice were maintained to support colony propagation and experimental animal production. Briefly, LDLR^fl/fl^ × LDLR^fl/fl^ and LDLR^fl/fl^ × LDLR^fl/-^) breeding were established to generate homozygous LDLR^fl/fl^ offspring. Because LDLR^fl/fl^ breeding pairs exhibited reduced reproductive performance, LDLR^fl/fl^ × LDLR^fl/-^ breedings were routinely incorporated to improve colony productivity while maintaining the desired genotype distribution.

Another breeding colony was maintained to generate LDLR^fl/-^;Cre^+/-^ mice. Homozygous CMV-Cre or Heterozygous LCK-Cre transgenic females were crossed with male LDLR^fl/fl^ mice to produce F1 hyrbid. All pups were genotyped to confirm CMV/LCK-Cre and/or LDLR genotypes before assignment to breeding or experimental cohorts.

For both colonies: Female breeders were introduced into breeding cages at 8–10 weeks of age and paired continuously with a single age-matched male through multiple litters. Pups were ear-tagged and tail biopsied for genotyping on postnatal day 21. Pups were weaned on postnatal day 28 to account for the strain’s smaller size and to organize weanlings by their confirmed genotype. Breeders were retired after weaning a fifth litter, following failure to produce a litter within 8 weeks of pairing or after a previous pregnancy, or if a litter was nonviable because of neonatal mortality or parental cannibalization.

### VSV-G lentivirus preparation

Lentivirus was generated through transfection of HEK293T cells. On the day before transfection, 3-4x10^6^ HEK293T cells were added to a 10cm dish with fresh D10 media. The next day, once the HEK293T cells were 70-80% confluent they were transfected by first incubating a mixture of 30μL of 1 mg/mL polyethyleneimine (PEI) (Polysciences, cat #23966-1), 1.6 μg pMD2.g, 3.3μg psPAX2, 5μg expression plasmid, and 1mL optimum at room temperature for 15 minutes before adding the mixture dropwise to the HEK293T cells. After 24 hours, the media was replaced. 48 hours after media replenishment the viral supernatant was collected, filtered with a 0.45 μM filter, and either directly aliquoted and stored at -80˚C or concentrated through ultracentrifugation at 21,800xg for 2.5 hours at 4 ˚C before being aliquoted and stored at -80˚C. For the studies outlined in this manuscript, the expression plasmids include a plenti-EF1α-TagBFP2 plasmid.

Viral titers were calculated based on transgene expression in Jurkat cells following transduction. On day 0 of the titer, a series of volumes of thawed lentiviral supernatant was added to wells with Jurkat cells and the cultures were mixed well. 48-hours later, transduction efficiency of the Jurkats was assessed by determining the percent of live cells expressing the transgene with flow cytometry using a Cytoflex 3- or 4-laser. Live cells were identified by staining samples with LIVE/DEAD Fixable Near-IR (ThermoFischer, catalog #L34975). Titers for the mCART19 lentivirus was calculated based on the percent of live cells expressing the myc-tag that is located at the N-terminus of the CAR construct. The myc tag was detected by staining samples with an anti-myc antibody (Cell Signaling Technology, cat #2233S).

### VSV-G LV gene delivery to mouse T cells

For the experiments outline in this paper, mouse splenocytes and T-cells were isolated from 6-12-week-old mice and grown *ex vivo* in mouse T-cell cell expansion media that was composed of RPMI 1640, 10% FBS, 1% Penicillin-Streptomycin, 1% NEAA (ThermoFischer, cat #11140050), 0.1% beta mercaptoethanol (ThermoFiscer, cat # 21985023), 1% sodium pyruvate (ThermoFischer, cat #11360070), and 1% insulin-transferrin-selenium (ITS) (ThermoFischer, cat #41400045). Fresh 10ng/mL mouse IL-2 (ThermoFischer, cat #212-12-100UG) was added to the media each time cells were passaged.

We first generated a single-cell suspension from the spleen and lysed the red blood cells with ACK lysis buffer (ThermoFischer, cat #A1049201). Then, when applicable, mouse T-cells were isolated from mouse splenocytes with the EasySep™ Mouse T Cell Isolation Kit (StemCell, cat #19851). Following isolation, mouse splenocytes and T-cells were stimulated by adding them to a tissue culture plate that was pre-coated with 0.5ug/mL mouse anti-CD3 antibody (2C11) (BioXCell, cat #BE0001-1) and 5ug/mL anti-CD28 antibody (37.51) (BioXCell, cat #BE0015-1). To induce hLDLR expression in mouse T-cells from LDLR^+/+^ mice, we pulsed 1μg/million CD5-conjugated Cre-lipid nanoparticles onto the T-cells at the same time that they were added to the αCD3/CD28-coated plate.

24-hours after activation on Day 1, mouse cells were mixed with lentivirus at the specified MOI (typically MOI = 10), 10ng/mL mouse IL-2, and 10μg/mL polybrene (Millipore Sigma, cat #TR-1003-G) and spun for 1.5 hours at 2000xg at 35 ˚C on a retronectin-coated plate (Takara Bio, cat #T100B). Then, 24-hours later, on Day 2, the media was replaced and the cells were transduced for a second time by following the same protocol. On Day 3, we removed the media and replaced it with fresh media with 10ng/mL IL-2. From Day 3-8, we continued to expand the T-cells by adding media with IL-2 to maintain a cell concentration of 1x10^6^ cells/mL.

### Verification of VSV-G LV integration into hLDLR^+^ mouse T cells

Genomic DNA was extracted using Qiagen DNeasy Blood & Tissue Kit from T cells on day 5 after LV gene delivery. DNA concentrations were measured using NanoDrop. The lentiviral plasmid used for BFP virus production served as the positive control. For each sample, 10 ng of genomic/plasmid DNA was used as the PCR template. PCR was performed using 2× LiTaq™ PCR Master Mix with an annealing temperature of 63 °C for 30 cycles. The PCR products were analyzed on a 1.5% agarose gel. Primers used for detecting the presence of EF-1α promoter: Forward – GGTCTTGAAAGGAGTGGGAATTGG; Reverse – CGGCCAGCTTGAGACTACC; Primers used for detecting the presence of BFP: Forward – CATCCCCGACTTCTTCAAGC; Reverse – GCTTGTGCCCCAGTTTGCTAG.

### Flow cytometry

All flow cytometry was performed on either the Cytoflex 3-laser or Cytoflex 4-laser in the Children’s Hospital of Philadelphia Flow Cytometry Core. Flow data was analyzed with either Kaluza Analysis 2.1 software or FlowJo v11 software.

Assessment of hLDLR Expression in Activated Mouse T-Cells: Expression of human LDLR was checked 24-hours following activation. Samples were stained with LIVE/DEAD Fixable Near-IR (ThermoFischer, catalog #L34975) and anti-human LDLR PE (ThermoFischer, cat #MA5-40996). The positive gate for hLDLR expression was determined by staining samples from an LDLR^+/+^ mouse that were not exposed the Cre-LNP.
Assessment of LDLR Expression in Activated Splenocytes: To evaluate hLDLR expression in LDLR^fl/-^;Cre^+/-^ mouse splenocytes, we first activated the isolated splenocytes on a CD3/CD28-coated plate for 24 hours *ex vivo*. Then, we stained samples with the following antibodies: LIVE/DEAD Aqua (ThermoFischer, cat #L34957), anti-mouse CD45.2 APC (BioLegend, cat #109814), anti-mouse CD3 APC-Cy7 (BioLegend, cat #100222), anti-mouse B220 PE-Cy7 (BioLegend, cat #103222), anti-mouse CD11b FITC (ThermoFischer, cat #11-0112-82), anti-mouse CD11c BV785 (BioLegend, cat #117336), anti-mouse NK1.1 PerCP/Cy5.5 (BioLegend, cat #108728), and anti-human LDLR PE (ThermoFischer, cat #MA5-40996). Before and during staining with antibodies, non-specific staining of immunoglobulins to Fc-receptors was blocked with the use of TruStain FcX™ (BioLegend, cat #101319). Activated splenocytes from wildtype C57BL6 splenocytes were similarly stained to determine positive gating for hLDLR expression. Unstained control samples were used to determine positive gating for other immune cell markers.
Gene delivery Efficiency Check: Transgene expression was repeatedly monitored following lentiviral transduction of mouse cells by using flow cytometry. Live cells were identified based on staining with LIVE/DEAD Fixable Near-IR (ThermoFischer, catalog #L34975). CAR expression was determined by staining samples with an anti-myc tag AF647 antibody (Cell Signaling Technology, cat #2233S). The positive gate for transgene expression was determined by using a stained untransduced control sample.

### Evaluation of transduction efficiency following *in vivo* expansion

To examine long-term transgene expression following lentiviral transduction of mouse splenocytes when grown in an *in vivo* setting, we injected 22-28-week-old NSG mice with 2x10^6^ Day 3 mouse splenocytes via tail vein injection, 24-hours after the second transduction. Eight days later, we collected the spleens from the mice that were transplanted, lysed the red blood cells with ACK lysis buffer (ThermoFischer, cat #A1049201), and performed flow cytometry on the splenocytes to assess BFP expression. The isolated splenocytes were stained with: LIVE/DEAD Aqua (ThermoFischer, cat #L34957), anti-mouse CD45 APC (BioLegend, cat # 103112), anti-mouse CD45.2 PE (BioLegend, cat #109808), anti-mouse CD3 APC-Cy7 (BioLegend, cat #100222), anti-mouse B220 PE-Cy7 (BioLegend, cat #103222), and anti-mouse NK1.1 PerCp/Cy5.5 (BioLegend, cat #108728). Before and during staining with antibodies, non-specific staining of immunoglobulins to Fc-receptors was blocked with the use of TruStain FcX™ (BioLegend, cat #101319). The transferred cells were identified as mouse CD45.2^+^ cells.

### Transduction and detection of VSV-G LV encoding hIL-2

Transduction with hIL-2 VSV-G LV was performed as described above. Eight hours after the second transduction, the culture medium containing viral supernatants was replaced with fresh medium. The medium was refreshed again after another 24 hours. Cell culture supernatants were collected after another 24 hours, and human IL-2 levels were measured using the ELISA MAX™ Deluxe Set Human IL-2 kit.

### Production of mRNA

mRNA sequences were purchased from RNA Technologies and Therapeutics, Montreal, Canada). In brief, codon-optimized sequences were cloned into an mRNA production plasmid containing optimized proprietary 5′ and 3′ untranslated regions (UTRs) and a 101-nucleotide poly(A) tail. Plasmid DNA templates were linearized and used for *in vitro* transcription (IVT) with T7 RNA polymerase in the presence of N1-methylpseudouridine-modified nucleosides and a CleanCap analog (TriLink) for co-transcriptional capping. Following DNase I digestion of the DNA template, the mRNA was purified using FPLC based chromatography to remove contaminants, then ethanol-precipitated, washed, and resuspended in nuclease-free water. The integrity and purity of the mRNA were verified by spectrophotometry, and capillary gel electrophoresis. Removal of double-stranded RNA was confirmed by dot blot and functional testing through transfection into human dendritic cells. Endotoxin levels were quantified using a chromogenic Limulus amebocyte lysate (LAL) assay; all assays tested negative.

### Preparation of mRNA containing unconjugated and targeted lipid nanoparticles

Ethanolic solutions comprised of SM-102/DSPC/Cholesterol/PEG (50/10/38.5/1.5 mole ratio, N:P 5.67) were rapidly mixed with an aqueous buffer solution containing mRNA in 25 mM sodium acetate (pH 4.0) using the NanoAssemblr™ Ignite™ formulation system. A flow rate ratio (FRR) of 3:1 and total flow rate (TFR) of 12 mL/min was used. LNPs used for targeting were prepared with the addition of DSPE-PEG2000 Azide. The resulting LNPs were then buffer exchanged against a sucrose-containing solution, concentrated using Amicon Ultra-15 mL Centrifugal Filter Units, sterile-filtered using 0.2-µm filters, and frozen until use. mRNA-LNPs were characterized as below: briefly, particle size was measured in PBS (pH 7.4) at 25 °C using dynamic light scattering (DLS) using a Malvern Zetasizer Ultra Red and a Malvern Nanoparticle Tracking Analysis systems (NS300), mRNA concentration and encapsulation efficiency were measured using a modified Quant-iT RiboGreen RNA assay. Briefly, duplicate wells of a black microplate received either TE buffer or TE containing 2% (v/v) Triton X-100 (TE/Triton). A mRNA standard curve was prepared for each of the two buffers. Plates were then incubated at 37 °C before adding RiboGreen (1:100 in TE buffer) to each well at a 1:1 (v/v) ratio. Fluorescence (Ex 485 nm/Em 530 nm) was measured afterward using the Tecan Spark^®^. Azide-functionalized LNPs were conjugated with to purified CD5 or IgG1 isotype control antibodies via click chemistry. DBCO-modified antibodies were clicked with LNPs overnight at 4C. Following incubation, antibody-decorated LNPs were purified by size exclusion on a Sepharose CL-4B column to remove unbound antibodies, concentrated using Amicon filters, and frozen until use. Particle size, mRNA concentration, and encapsulation efficiency were measured as described above.

### hLDLR induction in mouse T cells using anti-CD5 conjugated Cre-mRNA-LNPs

Mouse T cells and splenocytes were isolated from the spleens of the indicated mice. Cells were activated on anti-CD3/CD28-coated plates as described above. Anti-CD5-conjugated mRNA LNPs were added to the cells at 1μg (mRNA-equivalent)/million cells at the time of seeding. Cells were then monitored for hLDLR induction or transduced with VSV-G LV as described above.

## Data Availability

The original contributions presented in the study are included in the article/**Supplementary Material**, further inquiries can be directed to the corresponding author/s.

## Supplementary material

Supplementary Figure 1 Genotyping of the hLDLR transgenic mouse.

Supplementary Figure 2 Induction of hLDLR over time post mouse T cell activation. Isolated mouse T cells from WT or hLDLR^fl/-^CMV-Cre^+/-^ mice were activated on an αCD3/CD28 antibody-coated plate overnight. T cells were collected at the indicated times to assess hLDLR expression by flow cytometry. Each dot represents an individual mouse. Error bars represent standard deviation (n=3). *p<0.05, **p<0.01 by Student’s t-test.

Supplementary Figure 3 Lentiviral delivery of human IL2 into hLDLR^+^ mouse T cells. Total T cells isolated from hLDLR^fl/-^;CMV-Cre^+/-^ mice were activated, followed by two rounds of LV transduction. Culture medium was changed every 24 hours, the ELISA measurement of hIL2 secretion only used the culture supernatant collected in the last 24 hours. Error bars represent standard deviation (n=3). ***p<0.001 by Student’s t-test. Schematics were generated using BioRender.

Supplementary Figure 4 Characterization of LNP formation and mRNA encapsulation. LNP diameter and polydispersity index were determined by DLS, and the mRNA encapsulation was quantified by RiboGreen assay. Error bars represent standard deviation (n=3).

Supplementary Figure 5 Evaluation of hLDLR immunogenicity. Total T cells isolated from CD45.2^+^ hLDLR^fl/fl^ mice were activated for 24 hours with or without αCD5 Cre mRNA-LNP, followed by expansion for another 3 days, then adoptively transferred into CD45.1^+^ recipient mice (lymphodepletion preconditioning with 200mg/kg cyclophosphamide 24 hours before adoptive transfer). Seven days later, splenocytes were harvested from the recipient mice to monitor the persistence of donor T cells (CD45.2^+^ hLDLR^+^) by flow cytometry. Unless otherwise stated, all LNP treatments in this figure were performed using 1μg(mRNA-equivalent)/million cells. Each dot represents an individual mouse (n=5). Error bars represent standard error. ns, non-significant, by Student’s t-test. Schematics were generated using BioRender.
