# Supplementary material for: An inducible human LDLR transgenic mouse model for enhanced lentiviral gene delivery to murine immune cells
Source: Front Immunol. 2026 Oct 7;17:1941021. doi: 10.3389/fimmu.2026.1941021 (PMC13683734; doi:10.3389/fimmu.2026.1941021)
Supplement: Supplementary file 1 [file SupplementaryFile1.docx]

**Supplemental Figure 1**

**Figure S1. Genotyping of the hLDLR transgenic mouse.**

**Supplemental Figure 2**

**Figure S2. Induction of hLDLR over time post mouse T cell activation.** Isolated mouse T cells from WT or hLDLR^fl/-^CMV-Cre^+/-^ mice were activated on an αCD3/CD28 antibody-coated plate overnight. T cells were collected at the indicated times to assess hLDLR expression by flow cytometry. Each dot represents an individual mouse. Error bars represent standard deviation (n=3). * p<0.05, ** p<0.01 by Student’s t-test.

**Supplemental Figure 3**

**Figure S3. Lentiviral delivery of human IL2 into hLDLR^+^ mouse T cells.** Total T cells isolated from hLDLR^fl/-^;CMV-Cre^+/-^ mice were activated, followed by two rounds of LV transduction. Culture medium was changed every 24 hours, the ELISA measurement of hIL2 secretion only used the culture supernatant collected in the last 24 hours. Error bars represent standard deviation (n=3). *** p<0.001 by Student’s t-test. Schematics were generated using BioRender.

**Supplemental Figure 4**

**Figure S4. Characterization of LNP formation and mRNA encapsulation.** LNP diameter and polydispersity index were determined by DLS, and the mRNA encapsulation was quantified by RiboGreen assay. Error bars represent standard deviation (n=3).

**Supplemental Figure 5**

**Figure S5. Evaluation of hLDLR immunogenicity.** Total T cells isolated from CD45.2^+^ hLDLR^fl/fl^ mice were activated for 24 hours with or without αCD5 Cre mRNA-LNP, followed by expansion for another 3 days, then adoptively transferred into CD45.1^+^ recipient mice (lymphodepletion preconditioning with 200mg/kg cyclophosphamide 24 hours before adoptive transfer). Seven days later, splenocytes were harvested from the recipient mice to monitor the persistence of donor T cells (CD45.2^+^ hLDLR^+^) by flow cytometry. Unless otherwise stated, all LNP treatments in this figure were performed using 1μg(mRNA-equivalent)/million cells. Each dot represents an individual mouse (n=5). Error bars represent standard error. ns, non-significant, by Student’s t-test. Schematics were generated using BioRender.
